# A combined small RNA and transcriptome sequencing analysis reveal regulatory roles of miRNAs during anther development of Upland cotton carrying cytoplasmic male sterile *Gossypium harknessii* (D2) cytoplasm

**DOI:** 10.1186/s12870-018-1446-7

**Published:** 2018-10-17

**Authors:** Bingbing Zhang, Xuexian Zhang, Guoyuan Liu, Liping Guo, Tingxiang Qi, Meng Zhang, Xue Li, Hailin Wang, Huini Tang, Xiuqin Qiao, Wenfeng Pei, Kashif Shahzad, Chaozhu Xing, Jinfa Zhang, Jianyong Wu

**Affiliations:** 1 State Key Laboratory of Cotton Biology/Institute of Cotton Research of Chinese Academy of Agricultural Science, 38 Huanghe Dadao, Anyang, 455000 Henan China; 2 Department of Plant and Environmental Sciences, New Mexico State University, Las Cruces, NM 88003 USA

**Keywords:** Upland cotton, CMS-D2, miRNA-target pair, Restorer gene, High-throughput sequencing

## Abstract

**Background:**

Cytoplasmic male sterility (CMS) in flowering plants is usually caused by incompatibility between mitochondrial and nuclear genomes, and can be restored by nuclear genes known as restorer-of-fertility (*Rf*). Although the CMS/Rf system is useful and convenient for economic production of commercial hybrid seed, the molecular mechanisms of CMS occurrence and fertility restoration in cotton are unclear.

**Results:**

Here, a combined small RNA and transcriptome sequencing analysis was performed on floral buds at the meiosis stage in three-line hybrid cotton system, and differentially expressed microRNAs (DEMs) and their target genes were identified and further analyzed for a possible involvement in CMS and fertility restoration. Totally 10 and 30 differentially expressed miRNA-target gene pairs were identified in A-B and A-R comparison group, respectively. A putative regulatory network of CMS occurrence and fertility restoration-related miRNA-target pairs during anther development were then constructed. The RLM-RACE analysis showed that gra-miR7505b regulates a PPR gene (*Gh_D05G3392*) by cleaving precisely at the 643 nt and 748 nt sites. The further analysis indicated that the sequence variation in the binding regions of *Gh_D05G3392* and *Gh_D05G3356* may cause a lower cleavage efficiency of the PPR genes by miR7505b and miR7505 in R line, respectively, leading to the up-regulation of the PPR genes and fertility restoration. These results have established their genetic involvement in fertility restoration in the CMS-D2 system.

**Conclusion:**

Our combined miRNA and transcriptome analysis in three-line hybrid cotton system provides new insights into the molecular mechanisms of CMS occurrence and fertility restoration, which will contribute to further hybrid breeding in cotton.

**Electronic supplementary material:**

The online version of this article (10.1186/s12870-018-1446-7) contains supplementary material, which is available to authorized users.

## Background

Cotton (Gossypium spp.), one of the world’s most important crops, is a major source of natural fiber materials. Improving cotton cultivars is becoming critical to meet an increased industrial demand for more raw cotton and high-quality cotton fibers. Heterosis refers to the phenomenon in which a hybrid shows higher performance for a trait than both parents. Hybrid breeding, which efficiently exploits heterosis, is thus an important strategy in breeding programs to increase the yield and quality of many important crops, including rice, maize and cotton [1]. One of the major challenges in breeding for hybrid cotton, an often cross-pollinated crop, is controlling pollination. Artificial emasculation has been traditionally used in the production of hybrid seed for commercial cultivation [2, 3]. The recent application of a cytoplasmic male sterility/restorer-of-fertility (CMS/Rf) system, in which a maintainer line is used to confer a sterility trait in the corresponding CMS line through hybridization, has greatly aided the control of pollination for hybrid seed production in cotton [3–6]. Although many studies have focused on CMS and fertility restoration, the molecular mechanisms underlying these phenomena are still unclear.

CMS in flowering plants, a maternally inherited trait, is characterized by the absence of functional pollen grains [7] while the female gametes are still viable. CMS is usually caused by an incompatibility between mitochondrial and nuclear genomes. In most cases, the pollen function defect is attributed to the presence of an abnormal gene arising from rearrangements in the mitochondrial DNA. The products of the abnormal gene can disrupt normal mitochondrial functions such as supplying energy to meiosis and microspore and pollen development, thereby causing no pollen production or pollen to be non-functional [7–9]. Recently studies have revealed the molecular mechanisms involved in CMS in several crops, including rice [10–12], tobacco [13], radish [14], *Brassica juncea* [15] and pepper [16]. A study of CMS in cotton has demonstrated that *atp1* and *atp6* in CMS-D8 as well as *cox1* and *cox2* in CMS-D2 are putative CMS-associated genes [17]. Further study showed that nine nucleotides inserted in *atpA* gene in CMS-D2 line relative to its maintainer line may be involved in CMS occurrence in Upland cotton [18].

Loss of fertility in CMS plants, which exhibits normal vegetative growth, can be restored by nuclear genes known as *Rf* genes [19]. Fertility restoration generally requires the suppression of CMS-associated RNAs or proteins via the action of *Rf* gene product. Several *Rf* genes have been cloned in plants such as maize [20], petunia [21], radish [22, 23], rice [11, 24–26] and sorghum [27]. Except for maize *Rf2*, which encodes an aldehyde dehydrogenase that may be involved in the production of plant hormone indole-3-acetyl acetate [20, 28], most of the cloned *Rf* genes are reported to encode a pentatricopeptide repeat (PPR) protein. Additionally, the *Rf2* gene for Lead type CMS rice encodes a protein containing a glycine-rich domain [29]. Furthermore, the *Rf3* in maize CMS-S type affects the transcript of *orf355-orf77*, resulting in fertility restoration in sterile lines [30]. Two main CMS systems, CMS-D2–2 and CMS-D8, have been developed in cotton by transferring exotic cytoplasm from *G. harknessii* Brandegee (D2) and *G. trilobum* (DC.) Skovst. (D8) into Upland cotton (*G. hirsutum*, AD1) nuclear backgrounds [6, 18, 31]. Interestingly, *Rf1* gene from *G. harknessii* (D2) can restore the fertility of both CMS-D2 and CMS-D8, whereas *Rf2* gene from *G. trilobum* only restores CMS-D8 to male fertility [18, 31]. Although male sterile lines from both CMS systems does not produce pollen grains due to lack of normal meiosis, the *Rf1* and *Rf2* gene in cotton functioned sporophytically and gametophytically, respectively, and the two restorer genes are not allelic but tightly linked in 0.93 cM [18, 31]. Furthermore, a CAPS marker for *Rf1*-specific was developed based on a candidate PPR gene and could ensure the purity of restorer lines [32]. Wang et al. (2007) constructed a linkage map with 9 markers flanking the *Rf2* gene including a PPR-AFLP marker [19]. A differential display study between D8 restorer line (*Rf2rf2*) and its maintainer line (*rf2rf2*) showed that the down regulation of starch synthase explains the lack of starch accumulation in sterile *rf2* pollen grains in restored plants [6]. A transcriptome analysis between CMS and D8 restorer line using a gene chip showed that many CMS-associated genes are mainly related to cell wall expansion [5].

MicroRNAs (miRNAs), a class of endogenous small RNAs approximately 22 nucleotides in length, negatively regulate gene expression via mRNA cleavage or translational repression [33]. Many studies have indicated that miRNAs are involved during plant development in various biological processes, including leaf development, floral organ development, hormone signal transduction and biotic or abiotic stress response [34, 35]. MiRNAs have been identified and characterized in many crops during anther development, a highly programmed process in flowering plants. In *B. juncea*, 47 differentially expressed miRNAs between CMS and maintainer lines were identified that might participate in the regulatory network of CMS occurrence during anther development [36]. In *B. rapa*, 54 conserved and 8 novel miRNA families involved in pollen development [37]. Totally 162 known miRNAs have been identified in radish, of which 28 known and 14 potential novel miRNAs were found to be differentially expressed during anther development between CMS and maintainer lines [38]. In Upland cotton, six of 16 conserved miRNA families were revealed to be significantly differentially expressed between a genetic male sterile (GMS) mutant and wild type during anther development [39]. However, no results have been reported to identify miRNAs in relation to CMS in cotton.

Although numerous miRNAs associated with anther development have been identified in many crops, the molecular mechanisms underlying CMS occurrence and fertility restoration in three-line hybrid cotton systems are unclear. Specifically, there is a lack of reports on whether a candidate PPR gene for fertility restoration interacts with miRNA. To systematically investigate miRNAs and target-mediated regulatory network of CMS and fertility restoration in a three-line hybrid cotton system, we constructed small RNA libraries and transcriptome libraries from floral buds of near-isogenic CMS line (called A line, with the CMS-D2 cytoplasm and non-functional recessive restoring genes *rf1rf1*) and its maintainer line (called B line, with a normal fertile cytoplasm and *rf1rf1*) B and restorer line R line (called restorer line, with the CMS-D2 cytoplasm and functional dominant restoring genes *Rf1Rf1*) for the CMS-D2 based three-line hybrid cotton. Through the use of combined small RNA and transcriptome sequencing analyses, we aimed to explore the molecular mechanisms of CMS occurrence and fertility restoration during anther development in three-line hybrid cotton system. Our results should provide a valuable foundation for understanding of CMS in cotton and other crops.

## Results

### Cytological observation and analysis of anther development in CMS (A) line and its near-isogenic maintainer (B) line

**Fig. 1:**
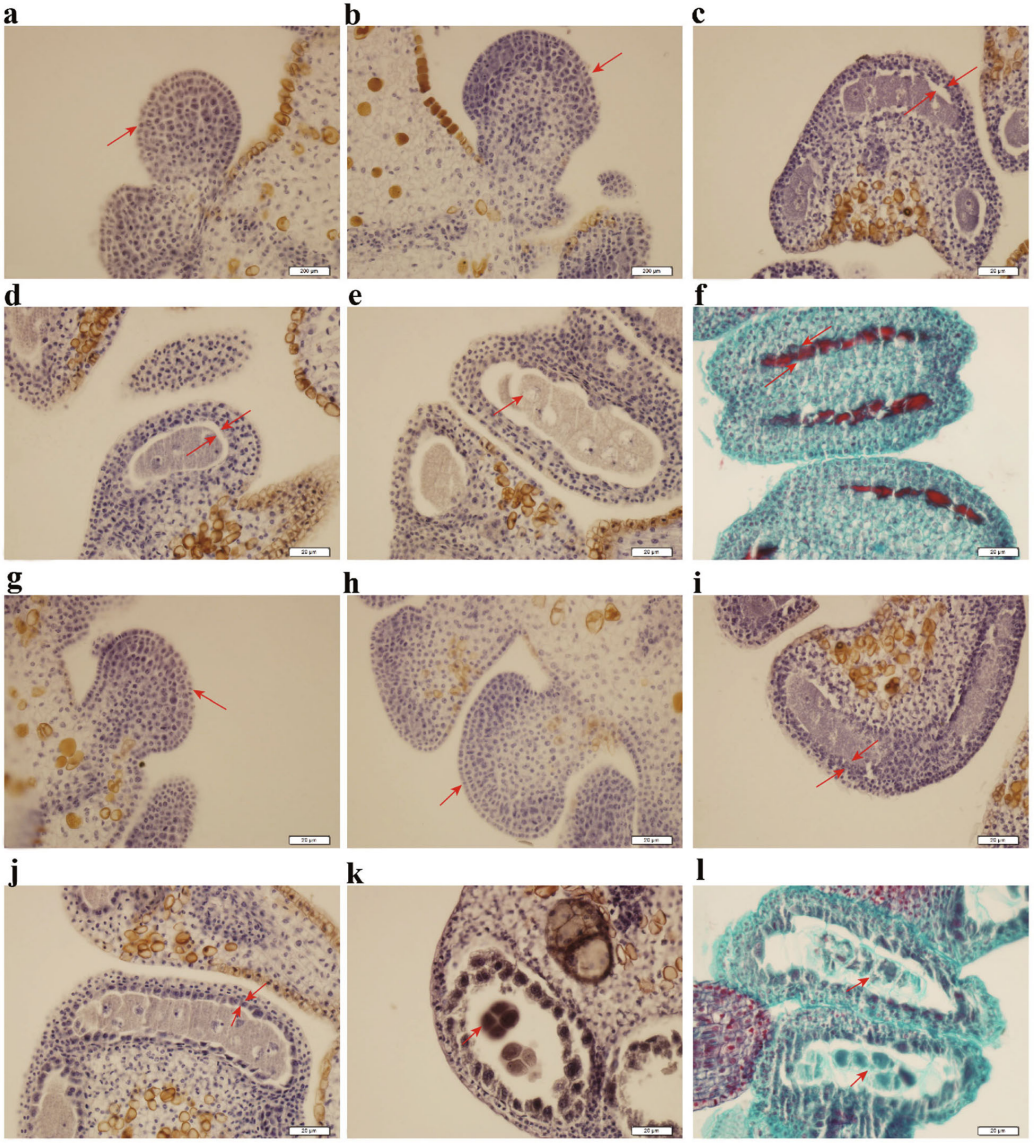
Cytological observation during anther development between A and B line of the CMS-D2 system in cotton. **a**-**e** The paraffin sections of floral bud for A line in 1–5 mm length, respectively. **f** Safranine and fast green dyeing for 5 mm floral bud of A line. **g**-**k** The paraffin sections of floral bud for B line in 1–5 mm length, respectively. **l** Safranine and fast green dyeing for 5 mm floral bud of B line

CMS-D2 is a sporophytic system. According to a genetic study, *Rf1* gene from *G. harknessii* (D2) can restore the fertility for both CMS-D2 and CMS-D8, and *Rf1* gene functioned sporophytically [31]. Mehetre (1982) showed that meiosis in cotton occurs at floral buds of ca. 3 mm in size [40]. For validation and sampling of floral buds for gene expression study at the right developmental stage, we compared the cytological characteristics during anther development (the floral bud from 1 to 5 mm) in CMS (A) line and maintainer (B) line of the CMS-D2 system. The paraffin sections did not show any significant difference when the floral buds at length from 1 to 2 mm between A and B line were compared. However, in the floral buds at length from 3 to 5 mm, the tapetum in A line was significantly smaller and the nucleus disappeared as compared with that in B line. Thus, consistent with previous reports [40], the CMS occurs during the floral bud growth stage when they are 3 mm in size (Fig. 1).

### Overview of transcriptome sequencing in near-isogenic CMS (A), maintainer (B) and restorer ® lines

To identify transcripts that are involved in CMS occurrence or fertility restoration, RNA-seq libraries from 3 mm floral buds of A, B, and R lines each with three biological replicates were constructed. Totally 9 RNA-seq libraries were constructed for sequencing. Totally 920,746,206 raw reads were obtained from these libraries (Table 1). After filtering out low-quality reads, 887,090,420 clean reads were used for alignment analysis to the reference TM-1 genome by using TopHat v2.0.9. As a result, a total of 59,939 out of 70,478 genes were detected.

**Table 1:** Data summary of transcriptome and small RNA sequencing in A, B, R lines

	Category	CMS (A) line (S(*rf1rf1*))	Maintainer (B) line (N(*rf1rf1*))	Restorer (R) line (S(*Rf1Rf1*))
Transcriptome	Raw reads	294,679,040	315,123,500	310,943,666
	Clean reads	283,884,614	303,319,558	299,886,248
	Valid ratio	96.337%	96.254%	96.444%
	Gene	59,939		
Small RNA	Raw reads	8,398,232	10,531,059	9,237,460
	Clean reads	5,972,568	6,453,537	5,991,695
	Unique reads	2,523,938	2,370,576	186,637
	Mapped sRNA	3,901,003 (65.32%)	4,364,067 (67.62%)	4,184,190 (69.83%)
	Known miRNAs	27,413	25,404	14,878
	Unannotated	1,572,274	1,566,417	1,327,258

The FPKMs (mean value of three biological replicates) of genes was further used to perform a differential expression analysis. For biological replicates, genes with a P-adjusted < 0.05 were assigned as being differentially expressed. Based on this analysis, totally 1913 genes showed significantly differential expression between A and B lines, of which 917 (47.9%) differentially expressed genes (DEGs) were up regulated and 996 (52.1%) DEGs were down regulated in the B line (Additional file 1: Table S1). In the A line vs. R line comparison, 3079 DEGs were up regulated and 1446 were down regulated in the R line. As compared to the B line, 2936 DEGs were up regulated and 1262 DEGs were down regulated in the R line.

Gene Ontology (GO) analysis was performed for all DEGs in the three comparison (i.e., A vs. B, A vs. R, and B vs. R) groups. Among the three comparisons, the A vs. B comparison identifies DEGs related to the male sterile cytoplasm; the A vs. R comparison identifies DEGs related to the functional restorer gene *Rf1* when it restores the male fertility in the CMS line; and the B vs. R comparison identifies DEGs related to *Rf1* under normal male fertile conditions. The results showed that the three comparison groups both contained the following terms: cell morphogenesis (GO:0000902) and cellular component morphogenesis (GO:0032989) (Additional file 1: Table S1). In addition, the Kyoto Encyclopedia of Genes and Genomes (KEGG) enrichment pathways for the DEGs in the three comparison groups, including protein processing in endoplasmic reticulum, alpha-linolenic acid metabolism, and fatty acid degradation pathway.

### Sequencing of small RNA libraries in A, B and R lines

**Fig. 2:**
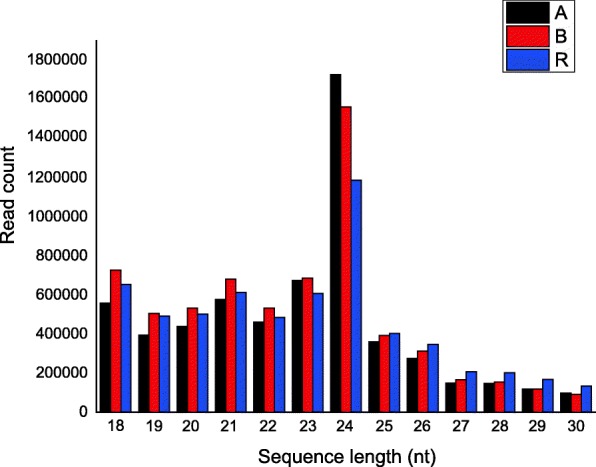
The distribution of small RNA length in three sample

Small RNA libraries representing A, B, and R lines were constructed, resulting in a total of 28,166,751 raw reads obtained. After removing low-quality reads, we obtained 5,972,568 clean reads ranging from 18 to 30 nt in length (2,523,938 unique reads) from A line, 6,453,537 (2,370,576 unique reads) from B line, and 5,991,695 (1,866,376 unique reads) from R line (Table 1). The most abundant sequences in small RNA libraries ranged between 18 and 24 nt, with 24 nt being most abundant (Fig. 2). After length filtration, the small RNA reads were mapped to the TM-1 reference genome. Reads of each line mapped to the reference genome accounted for 65.3% to 69.8% of the total reads, most of which were mapped to the positive chain of the TM-1 genome.

### Identification of known conserved miRNA families

**Fig. 3:**
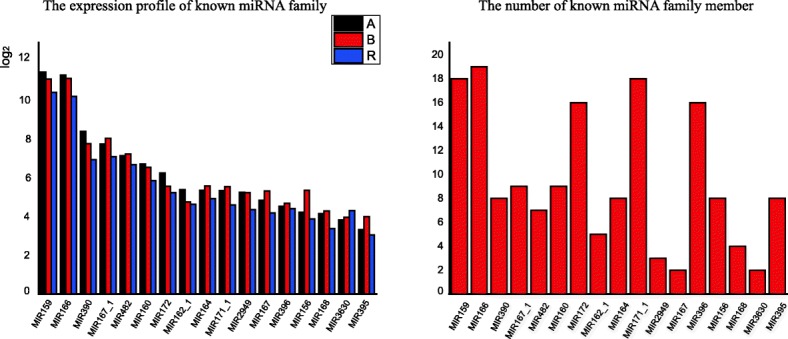
The expression profile and family member number analysis of part known miRNA family

To identify known miRNAs from three lines, the mapped small RNA reads were aligned to mature miRNA sequences and known miRNA precursors in miRBase 20.0. Only perfectly matched reads (with no mismatches) were identified as known miRNAs. A total of 370 known miRNAs representing 62 known conserved and 106 non-conserved miRNA families were identified (Additional file 2: Table S2). The distribution of miRNAs in different conserved miRNA families was analyzed. Most conserved miRNA families had more than three members. The miR166 family was represented by the most members (namely, 19), followed by miR159 and miR171–1 (each possessing 18 members) (Fig. 3). In contrast, some conserved miRNA families such as miR167 and miR2111 were represented by only one or two members.

In addition, the number of miRNA reads varied greatly with respect to conserved miRNA families. For example, miR159, miR166 and miR390 families were extremely abundant, whereas other miRNA families such as miR408 and miR530 had no expression in the A line. In addition, expression levels varied greatly between different members of the same miRNA family. An example is that, of the two members in the miR159 family, ath-miR159a displayed a high expression levels, whereas the expression of bdi-miR159a-3p was extremely scarce (Additional file 2: Table S2).

### Identification of novel miRNAs in A, B and R lines

Novel miRNAs and hairpin structures were predicted by combined miREvo and mirdeep2 software analyses, which uncovered 27 novel miRNAs in the three lines. These novel miRNAs were named temporarily and sequentially in the form of ghr-miR-number based on their chromosome locations. We also detected 27 miRNA* sequences (complementary to functional mature miRNAs), which was effective evidence of novel miRNAs (Additional file 3: Table S3). Among these sequences, the length distribution peak was 21 nt, representing the most abundant sequences (approximately 48.2%), a value consistent with the length distribution of miRNAs in other plants. The precursor length of these novel miRNAs varied from 54 to 291 nt, with an average length of 122 nt, similar to the 72 to 255 nt lengths of novel radish-anther miRNA precursors in a previous study (Zhang et al., 2016). The average minimum free energy (MFE) of the novel miRNA precursors was − 97.96 kcal/mol, with values varying from − 184.8 to − 59.4 kcal/mol. Most novel miRNAs were scarce compared with the majority of known miRNAs, and their expression levels varied sharply. For instance, ghr-miR-3 exhibited high and stable expression, whereas ghr-miR-8 and ghr-miR-26 displayed a low but variable expression across the three lines.

### Identification of miRNAs significantly differentially expressed among A, B and R lines

Based on the strict criteria (TPM ≥ 1 in any line, *P* ≤ 0.05 and |log_1.5_ (sample1/sample2)| ≥ 1), a total of 154 differentially expressed miRNAs (DEMs) (137 known and 17 novel) were identified by comparing the sterile A with fertile B and R lines (Additional file 4: Table S4). Totally 72 DEMs (65 known and 7 novel) were identified in A-B, of which 40 (38 known and 2 novel) and 32 (27 known and 5 novel) were up- and down-regulated in the A line, respectively, as compared to the B line. The expression levels of miRNAs also differed between members within a family: for example, gra-miR172b and ptc-miR172b-5p were down-regulated by 4.99- and 1.96-fold in the B line, respectively. Among the 126 DEMs in A–R, 12 (10 known and 2 novel) were up-regulated, and 114 (101 known and 13 novel) were down-regulated in the R line. Moreover, 10 and 9 miRNAs exhibited greater than 4-fold changes in expression levels in A–B and A–R, respectively. Interestingly, as compared to the A line, the two members of the miR477 family (mes-miR477a and nta-miR477a) that were the most significantly down-regulated miRNAs were down-regulated by 6.52- and 6.33-fold in the B and R line, respectively, whereas the most significantly up-regulated miRNA, i.e., ghr-miR-26, was up-regulated by 14.60- and 12.07-fold in these two lines.

Although the members of DEMs differed in A-B and A-R comparisons, 44 DEMs (39 known and 5 novel) were common in A-B and A-R comparisons, of which two cotton-specific miRNAs (ghr-miR7491, ghr-miR7500) were both up-regulated in A line as compared with the B and R line. However, 28 DEMs were only detected in A-B group, among which cotton-specific miRNAs ghr-miR2948-5p, ghr-miR2950 and ghr-miR7495a were all down-regulated in A line. In the A-R group, 82 DEMs were only uniquely detected, of which ghr-miR2949, ghr-miR3476, ghr-miR482 and ghr-miR7504 were all up-regulated in A line relative to R line.

**Fig. 4:**
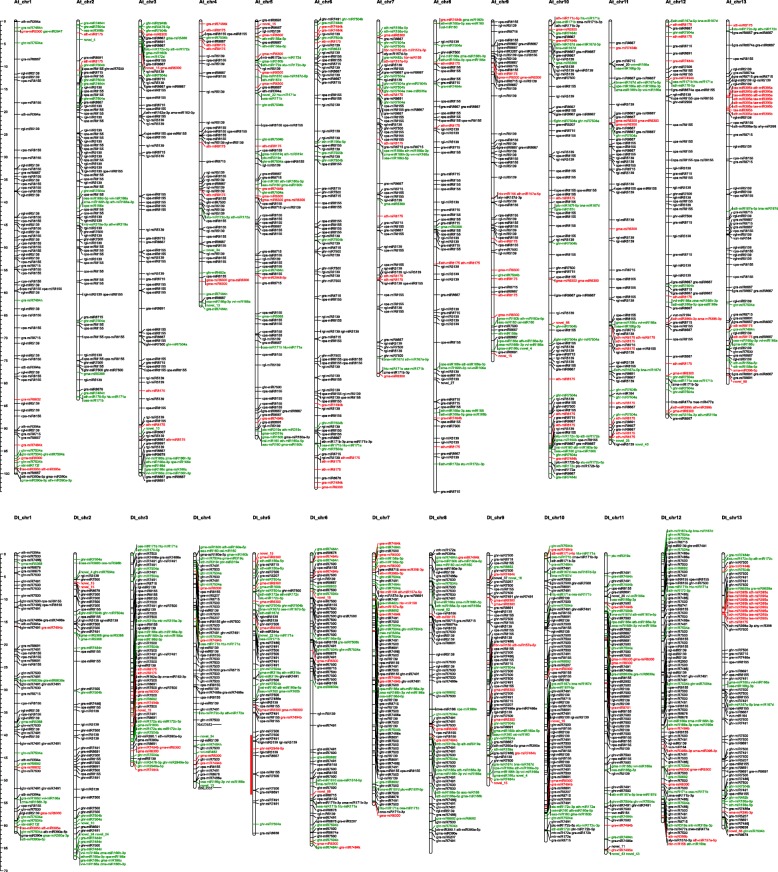
The putative chromosome location of differentially expressed miRNAs in A-B and A-R. The red color represent the DEMs only exist in A-B group, the green color represent the DEMs only exist in A-R group, the black color represent the DEMs common exist in A-B and A-R group. The red column near the Gh_D05 are the *Rf1* region by genetic mapping

Chromosome locations of miRNAs showed that most of the conserved miRNA families are widely distributed on different chromosomes (Fig. 4). For example, miR156 family was distributed on 11 chromosomes (A05, A06, A07, A09, A13 and D05, D06, D07, D09, D12, and D13), and miR160 family was also distributed on 11 chromosomes (A03, A05, A08, A09, A10, D03, D04, D05, D08, D09, and D10). Interestingly, more DEMs were distributed on the D subgenome than the A subgenome, due perhaps to the fact that miRNAs in the D subgenome have more critical roles in CMS occurrence or fertility restoration in cotton.

Previous studies indicated that *Rf1* gene is located on Gh_D05 chromosome, and genetic mapping indicated that the nearest SSR marker to *Rf1* was BNL3535 with 0.049 cM and NAU3652 on the other side with 0.078 cM [19, 32]. Interestingly, most DEMs located on Gh_D05 were down regulated in R line compared with A line. In the *Rf1* region flanked by the two SSR markers, 7 miRNAs (ghr-miR7500, ghr-miR7491, rgl-miR5139, ghr-miR2948-5p, gra-miR8667, cpa-miR8155 and gra-miR8691) were detected, among which, ghr-miR7500, ghr-miR7491, gra-miR8667, and gra-miR8691 were down regulated in R line, while rgl-miR5139, ghr-miR2948-5p, and cpa-miR8155 were up regulated in B line, as compared with A line. However, the target genes regulated by the 7 miRNAs did not show any significant difference in A-B or A-R comparisons, except for a protein kinase superfamily gene *Gh_SAPK2* (*Gh_A11G0474*) on chromosome A11 (regulated by ghr-miR7491) was up regulated in R line, as compared with A line. These miRNAs within the *Rf1* region did not show any sequence variations among the three lines, nor did their target genes. Furthermore, their target genes are not located in the *Rf1* region on Gh_D05. Therefore, we conclude that these 7 miRNAs and their targets are not genetically associated with CMS occurrence and fertility restoration.

Most importantly, 12 PPR genes were identified within the *Rf1* region of 0.127 cM between the two flanking SSR markers. However, only 5 PPR genes (*Gh_D05G3356*, *Gh_D05G3357*, *Gh_D05G3359*, *Gh_D05G3389*, and *Gh_D05G3392*) were up regulated in R line as expected for its functional and dominant *Rf1* allele, as compared with A and B line. While *Gh_D05G3357* gene only contains 84 amino acids, the remaining 4 PPR genes (*Gh_D05G3356*, *Gh_D05G3359*, *Gh_D05G3389*, *Gh_D05G3392*) belong to P-class PPR proteins and were selected as candidates for a further analysis. Interestingly, they are all targeted by gra-miR7505b, which is located on homeologous chromosomes Gh_A06 and Gh_D06. In addition, *Gh_D05G3356* gene is also targeted by ghr-miR7505, but with no difference in its expression level among A, B and R lines. Since *Rf1* is derived from its original CMS cytoplasm donor, i.e., *G. harknessii*, it is reasonable to believe that the *Rf1*-regulating small RNA gra-miR7505b is also carried on the D subgenome chromosome, i.e., Gh_D06. Among these 4 PPR genes, even though one or more may be directly responsible for the fertility restoration based on our prior high resolution mapping study [32], we speculate that there may be a special miRNA (i.e., miR7505b and/or miR7505) that regulates the expression of PPR- based *Rf1* gene in R line (see further analysis below).

### Target prediction and annotation of known and novel miRNAs

Because miRNAs regulate many developmental processes by mediating mRNA cleavage or translational repression [33], target prediction must be performed to understand the biological functions of miRNAs during anther development. In this study, we identified 4337 and 351 targets for 309 known and 23 novel miRNAs, respectively (Additional file 5: Table S5). A large proportion of predicted targets were genes for transcription-factor proteins, such as auxin response factors (ARFs), myb domain proteins, NAC (no apical meristem) domain transcriptional regulator proteins, basic helix-loop-helix (bHLH) DNA-binding family proteins, squamosal promoter binding proteins (SPL) and several functional gene family proteins such as PPR proteins and protein kinase families that may play a critical role in gene regulation of anther development.

**Fig. 5:**
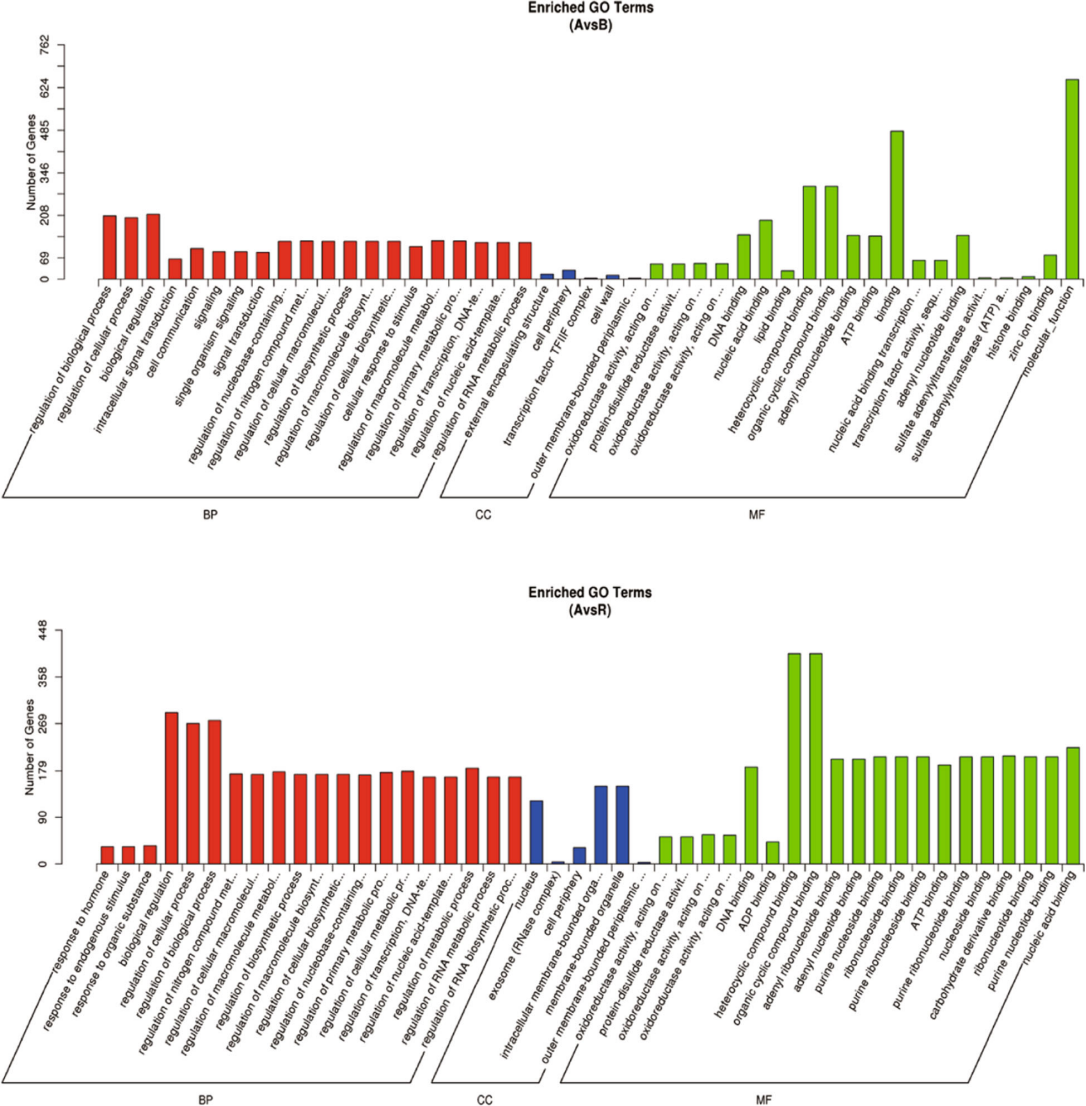
GO enrichment analysis of the predicted targets for DEMs between A-B and A-R

**Fig. 6:**
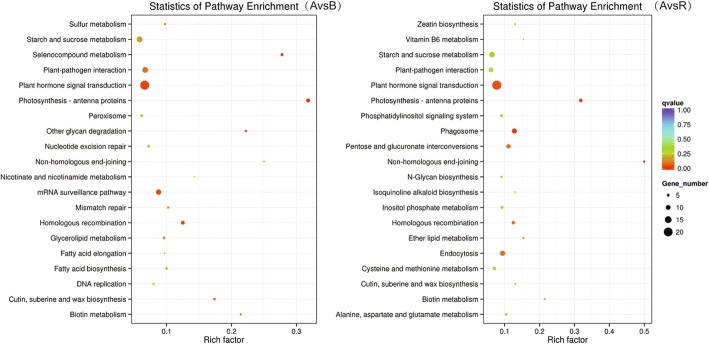
KEGG enrichment analysis of the predicted targets for DEMs between A-B and A-R

To further understand the biological functions of DEMs in A-B and A-R groups, we carried out GO and KEGG analysis of their target genes (Additional file 6: Table S6). In A-B group, 867 targets of 72 DEMs were assigned to 20 groups for the biological process category, 5 groups for the cellular component category, and 20 groups for the molecular function category (Fig. 5). The targets were concentrated on biological regulation, regulation of cellular processes, the cell periphery, and oxidoreductase activity and binding. The top three KEGG pathways were photosynthesis-antenna protein, selenocompound metabolism, and plant hormone signal transduction (Fig. 6). Within A-R group, 1107 targets of 126 DEMs were primarily concentrated on biological regulation, regulation of metabolic processes, membrane-bounded organelles, and organic cyclic compound binding. The top three KEGG pathways were photosynthesis-antenna protein, phagosomes, and plant hormone signal transduction. Overall, above GO terms and KEGG pathways may be associated with CMS occurrence and fertility restoration during anther development.

**Fig. 7:**
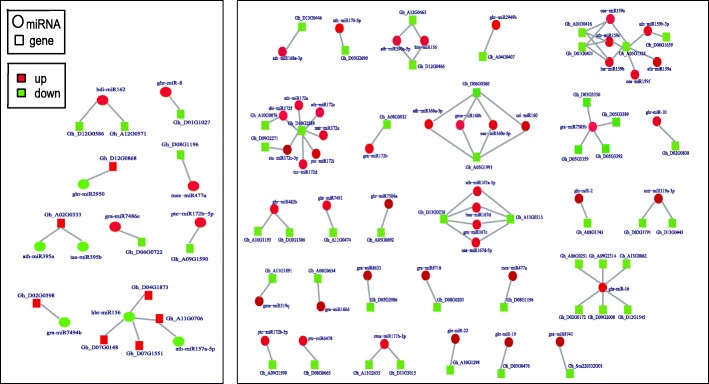
The summary of differential expressed miRNA-target pairs identified in A-B and A-R groups

To gain insights into the relationship between miRNAs and their targets, differentially expressed miRNA-target pairs were identified by comparing small RNA and transcriptome sequencing results. According to our screening criteria (FPKM ≥1.0 in any one line, *P* ≤ 0.05 and |log_1.5_ (sample1/sample2)| ≥1), 13 differentially expressed targets for 11 DEMs and 47 DEGs for 46 DEMs were identified in A-B and A-R comparisons, respectively (Fig. 7, Additional file 7: Table S7). These DEGs were associated with many transcription factors involved in gene expression regulation, signal transduction, and plant growth and development. Examples include SPL transcription factors targeted by the miR156 family, and NAC and ARF transcription factors targeted by miR164 and miR160 families, respectively. Additionally, many functional genes were identified as differentially expressed targets of miRNAs, such as 3-ketoacyl-acyl carrier protein synthase I (Gh_D08G1196) as the target of mes-miR477a, peroxisomal membrane family protein (Gh_D13G0446) as the target of ath-miR168a-3p, and S-adenosyl-L-methionine-dependent methyltransferase superfamily protein (Gh_A10G1193) as target of ghr-miR482b. Furthermore, most miRNAs had more than one possible target gene, while different miRNAs could regulate the same targets. For instance, in A-B, hbr-miR156 was the regulator of both SPL transcription factor (Gh_A11G0706) and LRR protein kinase (Gh_D07G1551), whereas ath-miR395a and tae-miR395b could regulate the expression of ATP sulfurylase (Gh_A02G0333). In A-R, both gma-miR160b and ath-miR160a-5p could regulate ARF17 (Gh_D06G0360), whereas gra-miR7505b was the regulator of four PPR genes.

### MiRNA–target pair expression pattern validation by qRT-PCR and RLM-RACE analysis

**Fig. 8:**
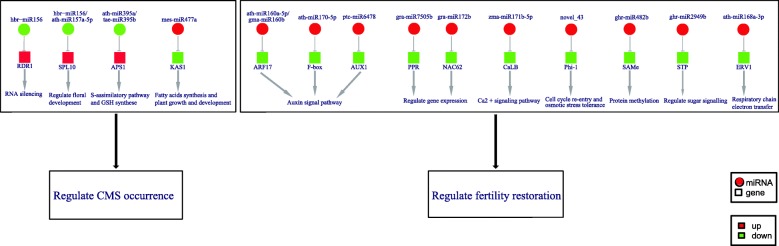
The putative miRNA-mediated regulatory network of CMS occurrence and fertility restoration

**Fig. 9:**
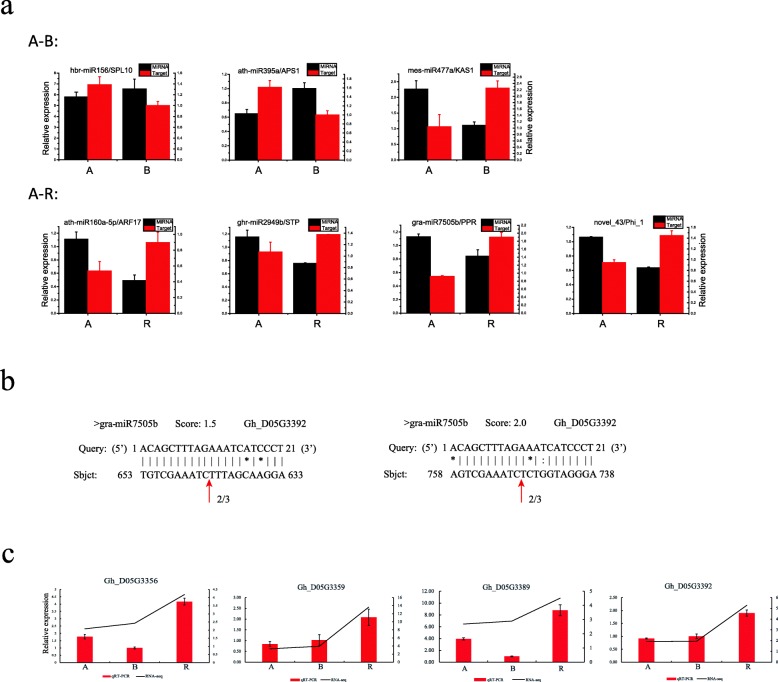
Validation of the expression patterns and cleavage site of miRNA-targets by qRT-PCR and RLM-RACE. **a** The black and red column represent the expression of miRNA and target respectively. A: sterile line, B: maintainer line, R: restorer line. **b** The red arrow represent the cleavage site by RLM-RACE. **c** The red column and black line represent the expression of qRT-PCR and RNA-seq respectively

To further narrow our analysis, we first constructed a putative regulatory network of miRNAs potentially functioning in CMS occurrence or fertility restoration during anther development in cotton (Fig. 8), based on the differentially expressed miRNA-target pairs identified in the A-B and A-R comparison groups. We then studied the dynamic expression patterns of miRNA-target pairs including three pairs (hbr-miR156-SPL10, ath-miR395a-APS1 and mes-miR477a-KAS1) in the A-B comparison group and four pairs (ath-miR160a-5p-ARF17, ghr-miR2949b-STP, gra-miR7505b-PPR and ghr-miR-10-Phi_1) in the A-R comparison group for validation using qRT-PCR (Fig. 9). The qRT-PCR results for all of the seven miRNA-target pairs were consistent with the RNA-sequencing data, with a negative correlation observed between the miRNA expression and the expression of corresponding target in different libraries. In the A-B group, for example, hbr-miR156 and ath-miR395a were up-regulated, while their corresponding targets SPL10 and APS1 were down-regulated in B line; and the down-regulated mes-miR477a also exhibited a negative correlation with its up-regulated target KAS1 in B line. In A-R, four miRNAs (ath-miR160a-5p, ghr-miR2949b, gra-miR7505b, and ghr-miR-10) were down-regulated, while their targets were up-regulated in R line.

To validate the cleavage sites, gra-miR7505b-PPR pairs were chosen for an RLM-RACE analysis. Similar to the bioinformatics prediction result, the RLM-RACE analysis showed that gra-miR7505b cleaves the PPR (*Gh_D05G3392*) gene precisely at the 643 nt and 748 nt sites. Both the expression of the miRNA-target pair and the RLM-RACE analysis indicate that this miRNA may participate in CMS occurrence or fertility restoration during anther development by silencing their corresponding targets (see next section for more analysis).

### Sequence variation analysis of the gra-miR7505b-PPR pairs in A, B and R lines

**Fig. 10:**
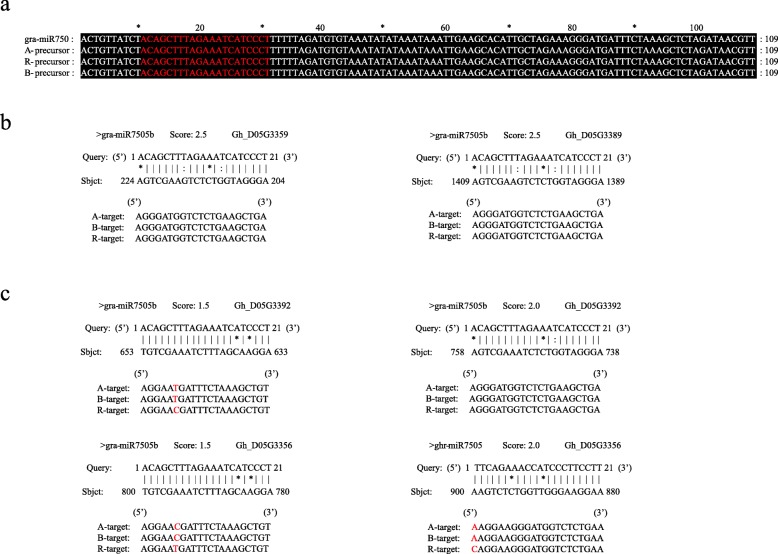
Comparison the sequence variation of gra-miR7505b-PPR pairs in A, B and R lines. **a** The sequence alignment of gra-miR7505b precursors. The red sequence represent mature sequence of gra-miR7505b. **b** The binding region sequence of *Gh_D05G3359* and *Gh_D05G3389* among the three lines. **c** The binding region sequence of *Gh_D05G3392* and *Gh_D05G3356* among the three lines. The red colour represent the sequence variation among the three lines

To understand if gra-miR7505b-PPR pairs are genetically associated with CMS and fertility restoration in the CMS-D2 system, we compared the sequences of gra-miR7505b and its four target PPR genes among A, B and R lines. As Fig. 10a shows, there was no difference in the mature sequences of gra-miR7505b among the three near-isogenic lines. For the four target PPR genes, no sequence difference was detected in the gra-miR7505b binding region of *Gh_D05G3359* and *Gh_D05G3389* among the three lines (Fig. 10b). However, for *Gh_D05G3392* gene with two binding sites for gra-miR7505b, no difference was detected in the binding region from 738 nt to 758 nt; however, in another binding region from 633 nt to 653 nt, the nucleotide in position 638 in R line was “C”, which was “T” in A and B line. This SNP produces a mismatch with gra-miR7505b in this position for R line. As a result, the cleavage efficiency of the miRNA on the PPR transcripts would be reduced in R line, resulting in its higher expression and then fertility restoration. On the contrary, the better binding between gra-miR7505b and the PPR target in A line would likely reduce the production of the PPR protein, leading to CMS in the sterile A line. For *Gh_D05G3356* gene also with two miRNA binding regions (at 780 nt to 800 nt for gra-miR7505b and at 880 nt to 900 nt for ghr-miR7505), the SNP (“C” in A/B to “T” in R line) at 785 nt caused a mismatch with gra-miR7505b in A and B line. In position 900 nt, a SNP (“C” in R line and “T” in A and B line) caused a mismatch with ghr-miR7505 in the 3′ end of its mature sequence in R line (Fig. 10c). Therefore, this PPR gene expression in R line may be suppressed by ghr-miR7505 but not by gra-miR7505b, as compared to A and B line for which the opposite was true. Overall, all the 4 PPR genes in R line were up-regulated as compared to A and B line. Previous studies have shown that SNPs in the binding regions of miRNA target genes reduce the miRNA affinity with the target sequence to lose its original regulation function, leading to expression changes of related genes [41, 42]. In this study, SNPs were detected in the binding regions of gra-miR7505b and ghr-miR7505 in their two target PPR genes, i.e., *Gh_D05G3392* and *Gh_D05G3356*, which may also affect the regulation function of the miRNAs, leading to changes in expression of the two PPR genes, i.e., decreased expression and male sterility in A line and increased expression and fertility restoration in R line.

## Discussion

Anther development is essential for pollen formation. The CMS-D2/Rf1 system can play a critical role in hybrid seed production, as it can potentially be exploited to reduce costs and ensure hybrid seed purity. Although several studies have been conducted to explore the genetic basis of CMS phenomena in crops, the molecular mechanisms of CMS occurrence and fertility restoration during anther development are unclear. High-throughput sequencing technology provides an efficient tool to explore the expression profiles of a large number of DEGs and DEMs related to CMS and fertility restoration. We therefore performed high-throughput small RNA and transcriptome sequencing analyses of floral buds from three-line cotton system to identify and characterize miRNA-target pairs involved in anther development. Our results should facilitate elucidation of roles of miRNA-target pairs in CMS occurrence and fertility restoration in cotton and other crops.

### Overview of small RNA sequencing

In this study, more than 18 million clean reads were obtained, with sequences between 18 and 24 nt being the most abundant. The most abundant length was 24 nt, followed by 21 nt. This result was consistent with sRNA distribution patterns reported in cotton [43], *Mesembryanthemum crystallinum* [44], *Eucommia ulmoides* [45] and *Medicago sativa* [46]. Interestingly, the most abundant sequence length in this study was 24 nt, and previous study also indicated that most siRNAs are 24 nt long [47]. Thus, siRNAs accounted for a large proportion of small RNA data of 3-mm floral buds in cotton.

### Gra-miR7505b, miR7505 and their PPR gene targets

In this study, the negative correlation between DEMs and targets allowed us to identify 13 DEGs for 11 DEMs in A-B and 47 DEGs for 46 DEMs in A-R by combining small RNA sequencing and transcriptome data. Moreover, a putative functional network of CMS occurrence or fertility restoration-related miRNA-target pairs during anther development was constructed as a basis for a further analysis. The most interesting finding was the identification of gra-miR7505b and its PPR gene targets. Previous studies have indicated that most *Rf* genes encode PPR proteins; for example, the *Rf4* gene for wild abortive-type CMS of rice encode a PPR protein to reduce CMS-causing orf352-containing transcripts to restore pollen fertility in restorer F1 plants [48]. The PPR protein Rf6 was found to function with hexokinase 6 to promote the processing of the aberrant CMS-associated atp6-orfH79 to rescue HL-CMS of rice [49]. In this study, the down-regulated gra-miR7505b was predicted to promote the up-regulation of PPR gene *Gh_D05G3392* in R line. Furthermore, a mismatch between the miRNA-binding region sequence and the gra-miR7505b may cause a lower level of recognition of target PPR gene by the miRNA, leading to higher expression of the PPR gene for fertility restoration in R line. Another interesting result was the identification of another miR7505b-targeted PPR gene *Gh_D05G3356*. Its two miR7505 binding sequences may have an opposite effect in regulating the expression of the PPR gene in that the sequence variation in the binding region of gra-miR7505b may cause a better binding in R line, while the sequence variation in binding region of ghr-miR7505 may cause a poor binding due to a mismatch with the 3′ end of the mature sequence of ghr-miR7505 in R line. Thus, both the down-regulation of miR7505b and its the sequence variation in the binding region would cause miR7505 not efficiently cleavage *Rf1*-associated PPR gene transcripts, leading to the up regulation of the PPR gene needed for normal anther development and fertility restoration in R line. This study has identified miR7505b and miR7505 as important regulators of PPR genes in relation to fertility restoration in CMS-D2 cotton for the first time. However, whether either or both miRNAs function independently or in concert needs further detailed studies in the future.

### Other differentially expressed miRNA-targets pairs among A, B and R lines

Several other miRNA-target pairs are also related to floral and pollen development, however, these miRNA-target pairs did not lie within the same genetic interval on chromosome Gh_D05 as the *Rf1* locus. As shown in the network, many important transcription factors and functional genes were associated with floral organ development, signal transduction and organellar gene expression. One such example is SBP-box genes, a transcription factor family with critical functions in plant growth and development. A previous study has demonstrated that SPL3 is targeted by miR156 to regulate the timing of flower formation [50]. Furthermore, multiple *SPL* genes targeted by miR156 are necessary for the formation of fully fertile flowers [51–53]. In our study, hbr-miR156 was down-regulated while SPL10 was up-regulated in A relative to B line; this suggests that the reduced expression of hbr-miR156 enhanced the expression of SPL10 that may regulate CMS occurrence.

A negative correlation was also uncovered between the expression of ath-miR395a and its target, APS1. A previous study has indicated that APS is the key enzyme in the sulfur-assimilatory pathway, which has recently been found to regulate glutathione (GSH) synthesis [54]. In cotton, miR395-APS1 is involved in salt and drought stress response [55]. In the present study, ath-miR395a was down-regulated while its target APS1 was up-regulated in A line. This trend is consistent with that observed in *B. juncea* [36], which suggests that the miR395-APS1 pair is associated with CMS occurrence in that miR395 presumably regulates APS1 to promote an increase in GSH expression to suppress the oxidative stress in A line.

Ath-miR160a-5p was predicted to target the ARF17 transcript. Several studies has indicated that ARFs are key regulators of gene expression response to auxin and floral organ formation [53, 56, 57]. The expression level of ath-miR160a-5p was down-regulated in R line, whereas ARF17 was up-regulated, which suggests that the up-regulation of ARF17 plays important roles in regulating normal anther development in R line.

The cotton-specific ghr-miR2949b was predicted to act as a regulator of sugar/nucleotide transporter protein (STP). According to a previous study, pollen of CMS lines lacks soluble sugars [58], with most carbohydrates in pollen tissue transported from other tissues, usually via STPs [59]. Our results revealed that down-regulation of ghr-miR2949b increased STP expression levels in R line. Consequently, ghr-miR2949b may promote normal anther development in R line by regulating STP expression.

However, it is important to recognize that the above miRNA-target pairs do not have a direct genetic link to fertility restoration for the CMS-D2 system, i.e., there is no genetic causative relationship between them and fertility restoration, because none of them is located within the *Rf1* gene region on chromosome D5. Further studies should be focused on how some of these miRNA-target pairs are molecularly regulated by PPR genes, leading to pollen development and male fertility restoration.

## Conclusions

In this study, we attempted to reveal the molecular mechanisms involved in CMS occurrence and fertility restoration by combining small RNA and transcriptome sequencing of floral buds among CMS, maintainer, and restoration lines. Among several differentially expressed miRNA-target pairs in A-B and A-R groups identified for the first time, the miR7505b/miR7505 and two PPR gene targets are genetically related to fertility restoration in CMS-D2 system. Our new findings will serve as a valuable foundation to shed light on the miRNA-mediated regulatory network of CMS occurrence and fertility restoration.

## Methods

### Plant materials

The CMS-D2 three-line hybrid cotton system was developed at the Cotton Research Institute (CRI), Chinese Academy of Agricultural Science (CAAS). In previous study, the CMS line with the CMS-D2 cytoplasm was crossed with the restorer line, and the maintainer line with normal fertile Upland cotton (AD1) cytoplasm as recurrent male parent to backcross with the F_1_ plants to construct a BC_8_F_1_ population. From this segregating population, the sterile and fertile plants were selected as the CMS-D2 A line and restorer line ®, respectively [32]. The CMS line (A) is homozygous for the recessive (i.e., nonfunctional) fertility restorer alleles S(*rf1rf1*), while maintainer line (B) harbors normal fertile Upland cotton cytoplasm and has the same nuclear allelic composition N(*rf1rf1*). The restorer line ® is homozygous for dominant (i.e., functional) fertility restorer alleles S(*Rf1Rf1*) to allow recovery of fertility in CMS-D2 cotton plants in a cross of A × R. The three lines were planted under normal production conditions. For sample collection as described in previous studies [5, 18], each genotype was grown side by side in field, and floral buds approximately 3 mm in length (corresponding roughly to the stage of male meiosis) were collected and combined from about 100 plants (one floral bud was collected from one plant) with three independent biological replicates. All collected floral buds were cut above ovaries and immediately frozen in liquid nitrogen after harvesting and stored at − 80 °C before use.

### High-throughput small RNA and transcriptome sequencing

Extraction of total RNA was performed using Spectrum™ Plant Total RNA kit according to manufacturer’s instructions. Equal amounts of RNA from three biological replicates were used to construct small RNA libraries (A, B and R) and transcriptome libraries (A1–3, B1–3 and R1–3). Small RNA libraries were generated as follows: 3′ and 5′ adaptors were ligated to small RNA, with first-strand cDNA then synthesized using M-MuLV reverse transcriptase (RNase H^−^) followed by PCR amplification. Fragments corresponding to 140 to 160 bp (the length of small RNA plus the 3′ and 5′ adaptors) in the PCR product mixture were separated and purified by 8% polyacrylamide gel electrophoresis. The transcriptome libraries were generated using a NEB Next Ultra RNA Library Prep kit for Illumina following manufacturer’s recommendations. Both small RNA and transcriptome sequencing were performed on an Illumina Hiseq 2500/2000 platform.

Clean reads were obtained from the raw reads by removing low-quality reads, 5′ adaptor contaminants, reads containing poly-N, A, T, G or C. Clean reads comprising 18 to 30 nt were mapped to the TM-1 reference genome using Bowtie [60, 61]. Only reads without mismatches were retained for expression and distribution analyses. Any small RNAs matching protein-coding genes, repeat sequences, rRNAs, tRNAs, snRNAs or snoRNAs were removed. The remaining small RNAs were aligned with known miRNAs in miRBase (http://www.mirbase.org/index.shtml) without mismatches to identify known/conserved miRNAs. The remaining unknown reads were used to predict novel miRNAs using miREvo [62] and mirdeep2 software [63]. The mRNA transcriptome was aligned to the TM-1 reference genome (http://www.cottongen.org) using TopHat after obtained clean reads. The raw sequence data of transcriptome and small RNA can be found in the National Center for Biotechnology Information (NCBI) under accession number SRX3421007 and SRX3422274, respectively.

### Differential expression analysis of miRNAs and mRNAs from A, B and R lines

MiRNA expression levels were normalized based on transcripts per million (TPM) using the following formula [64]: normalized expression = mapped read count / total reads × 1,000,000. The criteria used to define significant differential expression of a miRNA between two samples were fold change = |log_1.5_ (sample1/sample2)| ≥ 1 and *P* ≤ 0.05. For transcriptome, gene expression were computed by summing the FPKMs of transcripts in each sample using Cuffdiff v2.1.1 [65], and the mean FPKMs from three biological replicates were used for differential expression analysis, and genes with an adjusted *P*-value < 0.05 between two lines were assigned as differentially expressed.

### Prediction and annotation of targets of identified miRNAs

Candidate targets of miRNAs were predicted with psRNATarget (http://plantgrn.noble.org/psRNATarget/). GO enrichment analysis was performed with GOseq package [66] and KEGG enrichment analysis was tested using KOBAS software [67].

### Quantitative (q) RT-PCR validation of DEM and DEG expression

QRT-PCR was performed to evaluate the validity of transcriptome and small RNA sequencing results. MiRNAs and total RNAs were extracted from floral buds and reverse transcribed to cDNA using TransScript miRNA First-Strand cDNA Synthesis SuperMix kit (TransGen, Beijing) and PrimeScript RT reagent kit (Takara, Dalian), respectively, following the manufacturer’s guidelines. MiRNA-specific primers were designed according to the mature miRNA sequence (Additional file 8: Table S8). For qPCR, reactions were performed in 20-μL volumes containing 1-μL diluted cDNA, 10-μL 2× SYBR Green Mix (Takara), miRNA-specific primers and universal primer. The amplifications were carried as follows: 94 °C for 30 s, then 40 cycles of 94 °C for 5 s, 55 °C for 15 s and 72 °C for 25 s. *Ubiquitin 6* (*GhUBQ6*) (Forward primer: CATTTCTCGATTTGTGCGTGTC; Reverse primer: GGGGACATCCGATAAAATTGG) was reference gene for normalization. All reactions were performed with three biological replicates, and miRNA relative expression levels were calculated using the 2^−ΔΔCt^ method.

### RLM-5’ RACE and sequence variation analysis for gra-miR7505b-PPR

To validate the cleavage sites, RLM-5’ RACE was accomplished by using First Choice RLM-RACE kit (Ambion, USA), following the manufacturer’s guidelines. Gene-specific 5′ primer and 5’ RACE gene-specific nested primer were designed to amplify the cleaved transcripts. The 5’ RACE products were cloned with pEASY-T1 vector (TransGen, Beijing) and sequenced. Additionally, specific primers were designed to clone targets for sequence analysis. The PCR products were linked with pEASY-T1 vector and selected 8 clone in every sample to sequence. The MEGA7 was used for sequence alignment. All primers used above are listed in Additional file 8: Table S8.

## Additional files

Additional file 1: **Table S1**. Information of differentially expressed genes in A, B, and R lines. (XLSX 488 kb)

Additional file 2: **Table S2.** Information of conserved and non-conserved miRNA family in A, B, and R lines. (XLSX 24 kb)

Additional file 3: **Table S3.** Information of novel miRNA in A, B, and R lines. (XLSX 12 kb)

Additional file 4: **Table S4.** Information of differentially expressed miRNAs in A-B and A-R compare group. (XLSX 19 kb)

Additional file 5: **Table S5**. Annotation of predicted target genes for known and novel miRNAs. (XLSX 661 kb)

Additional file 6: **Table S6**. GO and KEGG analysis of predicted target genes for DEMs in A-B and A-R compare group. (XLSX 403 kb)

Additional file 7: **Table S7.** Information of differentially expressed miRNA-target pairs in A-B and A-R compare group. (XLSX 12 kb)

Additional file 8: **Table S8.** Primers for quantitative RT-PCR and RLM-5’ RACE and sequence variation analysis. (XLSX 9 kb)

## Abbreviations

A: CMS line
B: Maintainer line
CMS: Cytoplasmic male sterility
DEGs: Differentially expressed genes
DEMs: Differentially expressed microRNAs
FPKM: Fragments per kilobase of exon per million fragments
GO: Gene Ontology
KEGG: Kyoto Encyclopedia of Genes and Genomes
R: Restorer-of-fertility line
Rf gene: Restorer-of-fertility gene
RLM-RACE: RNA Ligase Mediated Rapid Amplification of cDNA Ends
